# Clinical Assessment of Thermotherapy Applications during Hepatectomy and Laparotomy in Sturgeon (*Acipenser ruthenus*): Impact on Bioparameter Variations Based on Liver Condition

**DOI:** 10.3390/vetsci10120682

**Published:** 2023-12-01

**Authors:** Gyoungsik Kang, Won-Sik Woo, Kyung-Ho Kim, Ha-Jeong Son, Min-Young Sohn, Hee Jeong Kong, Young-Ok Kim, Dong-Gyun Kim, Eun Mi Kim, Eun Soo Noh, Chan-II Park

**Affiliations:** 1 Department of Marine Biology and Aquaculture, College of Marine Science, Gyeongsang National University, Tongyeong 53064, Gyeongsangnam-do, Republic of Korea; sik27290@gnu.ac.kr (G.K.);; 2 Biotechnology Research Division, National Institute of Fisheries Science, Busan 46083, Republic of Korea; heejkong@korea.kr (H.J.K.);

**Keywords:** hepatectomy, laparotomy, sturgeon, fish surgery, sturgeon histology, fish suturing, thermotherapy

## Abstract

**Simple Summary:**

Surgical techniques in aquaculture are gaining attention. This study investigated the effects of high temperature on wound healing, hematopoietic recovery, and histological changes in sturgeon undergoing surgical procedures. The liver condition was found to play a pivotal role in the analysis. High temperature facilitated wound healing, but excessive induction of physiological activity caused damage. Further research is needed to promote animal welfare in fishery products.

**Abstract:**

Surgical techniques are gaining attention for treating physical diseases in aquaculture and aquarium fish. Sturgeon is a suitable species for surgical experiments due to its industrial significance. Maintaining homeostasis is crucial during surgical procedures, and the liver plays a major role in immune regulation. High temperature is suggested to improve physiological activity and wound healing. This study investigated differences in hepatectomy sturgeons’ tolerance and histopathological responses of internal organs. Moreover, this study investigated the effects of high temperatures on wound healing and hematopoietic recovery in fish undergoing surgical procedures. The liver condition was found to play a pivotal role in the analysis, and cortisol levels were affected by anesthesia. The results showed that high temperature facilitated hematopoietic recovery and wound healing, but excessive induction of physiological activity caused damage. Managing high temperatures and liver conditions induced a remarkable improvement in wound healing. However, anesthesia itself can be a significant stressor for fish, and wound healing requires a greater amount of energy. Further research is needed to understand the stress factors caused by surgical procedures and anesthesia and to promote animal welfare in fishery products.

## 1. Introduction

In recent times, the aquaculture industry has been focused on enhancing fish welfare and promoting the development of the personal aquarium market, while simultaneously increasing production [1]. During the culturing period of fish, a plethora of diseases have been observed, which are associated with physical, infectious, and environmental factors [2,3]. Aquaculturists are advised to prioritize disease control to minimize mortality in the event of an outbreak, and numerous researchers have endeavored to provide effective treatment methods [4]. However, unlike infectious and environmental problems, which can be resolved through chemotherapy and environmental improvement, respectively, physical injuries are difficult to cure due to the osmotic challenges that arise from aquatic living conditions [5]. As a result, the importance of new surgical techniques for the aggressive treatment of physical diseases in both aquaculture and aquarium fish is gaining attention among researchers [6]. Some researchers have suggested the availability of surgical techniques as an important aspect of treatment [7,8].

Surgery is a useful tool in the treatment of various diseases that require surgical intervention in fish [9]. For instance, basic surgery can effectively treat physical conditions such as intussusception, ulcers, and tumors [10,11,12]. Although laparotomy is a fundamental technique used in many surgical procedures for both humans and vertebrates, the study of fish surgery remains relatively scarce compared to that of humans and other vertebrates [7]. Nevertheless, recent studies on the surgical operation of fish have been increasing [13]. Furthermore, many researchers emphasize the necessity of histopathological analysis to investigate the relationship between wound healing and the microscopic structures of organs [14].

In this context, sturgeon is a suitable fish species for conducting surgical experiments due to its characteristics of harvest and industrial significance [15]. Sturgeons have been subjected to abdominal laparoscopic surgery for sex identification, as they have a long reproductive cycle and low spawning rates, making it difficult to distinguish between males and females [16]. Sturgeon, one of the most widely cultivated freshwater fish, is often slaughtered for its fine food ingredients such as caviar and/or its meat [17]. As the sturgeon takes a long time to reach maturity and has a low spawning rate, the roe is extracted from mature fish through a caesarian section, and then it is stitched back together to enable further spawning [18]. Additionally, rearing sturgeon until they reach marketable size in a short period is challenging, and satisfying the demand for sturgeon food ingredients within a short period is difficult [19]. In laparotomy and laparoscopic operations, many sturgeons have died due to infectious diseases, shock, unstable homeostasis, or accidents [19]. Therefore, it is crucial to maintain the physiological activity of the fish before performing any surgical procedures [7].

Maintaining homeostasis is crucial during surgical procedures [7]. The liver is a major organ responsible for homeostasis and immune regulation [20]. Many researchers suggest that maintaining healthy immunity and optimal physiological activity can aid in disease prevention [5]. Physiological activity is regulated by temperature, and high temperature has been used in human medicine to improve physiological activity and treat certain injuries [21]. Improving physiological activity can lead to more rapid wound healing compared to a depressed state [19]. Therefore, this study focuses on the differences in hepatectomy patients’ tolerance and the histopathological responses of internal organs, as well as investigating whether high temperatures can provide advantages in wound healing. Furthermore, the present study observed histological changes in the liver during the recovery period following the application of surgical procedures, considering the crucial role the liver plays in maintaining homeostasis.

## 2. Materials and Methods

### 2.1. Fish

A total of forty-eight individuals of the sterlet sturgeon species (*Acipenser ruthenus*) were obtained from a farm situated in Incheon, Republic of Korea. Before being allocated to individual experimental aquariums, the specimens were housed in a domestication tank for a period of four weeks, during which they were fed commercial diets (Propond Sterlet, JBL GmbH & Co. KG, Neuhofen, Germany). Further elaboration of the experimental parameters can be found in Table 1. The experiment was conducted in a 200 L glass tank equipped with a recirculating filtration system capable of filtering 15 L per hour. The water temperature was maintained at 18 °C until the induction of the initial anesthesia.

To assess the health status of the experimental fish, the condition factors (CF) were calculated using the following formula [22,23]:

Condition factor (CF) = Body mass (g)/{Total body length (cm)}^3^ × 100

The commencement of the experiment was defined as the moment following the first anesthesia, during which blood sampling, hepatectomy, and laparotomy procedures were performed. At the initiation of the first anesthesia, blood sampling was conducted simultaneously across all groups, followed by laparotomy and hepatectomy (with excised liver samples also utilized for analysis). Final sampling took place four weeks after the experiment’s commencement, involving six sturgeons sampled from each group. All samples were labeled with identifiable tags on their fins, enabling the observation of hematological and histological changes specific to each individual.

At the initiation stage of the experiment, blood sampling and surgical procedures were conducted at the initial rearing temperature of 18 °C. During the final stage of the experiment, manipulations were performed according to segment-specific water temperatures (18 °C and 28 °C). The 28 °C group gradually increased the water temperature at a rate of 1 °C per hour over a period of 10 h, starting immediately after the initial manipulation.

### 2.2. Anesthesia and Euthanasia

Except for the negative control group, all experimental groups were anesthetized with a concentration of 200 ppm MS-222 (Sigma-Aldrich, St. Louis, MO, USA) [2,6], and oxygen was supplied at 30 s intervals through a 3 mL syringe during the surgical procedure. The oxygen supply was administered by the assistant in a controlled manner, utilizing a syringe to inject water directly into the gills, following a predetermined schedule during the experiment.

The duration of anesthesia, handling time, and recovery time were measured for each individual, with anesthesia being based on the cessation of operculum movement and recovery being based on the point at which normal swimming behavior resumed in the tank.

Euthanasia was administered at the same concentration, and sampling was carried out after complete anesthesia was achieved.

### 2.3. Hepatectomy

**Figure 1:**
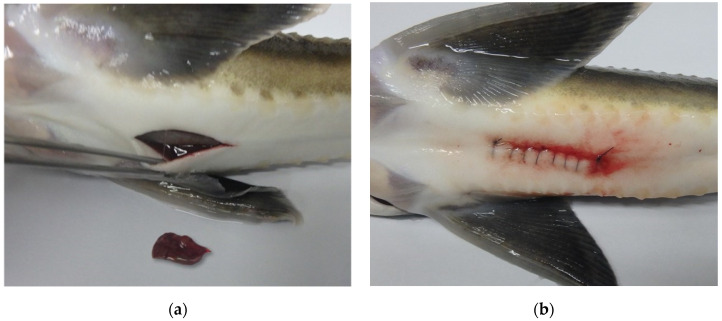
An image of the abdomen of a sturgeon with the liver excised and sutured is presented: (**a**) shows the appearance of the excised liver and (**b**) depicts a simple running suture.

The hepatectomy procedure was executed by employing a simple continuous suture technique (utilizing Blue nylon, 1 metric, Ailee, Republic of Korea), which involved excising roughly 0.1–0.3 g (approximately 10–15% of the total liver mass) of liver tissue per sample (Figure 1). The surgery was performed simultaneously with the initiation of the experiment, and following suturing, the incision site was treated with iodine, fusidate sodium, and Vaseline (Vaseline^®^, Unilever, London, UK) to prevent infection. The incision site size for hepatectomy and laparotomy was uniformly maintained at approximately 3 cm to ensure consistency. Minimal blood loss due to separate hemostasis and surgical procedures was observed, and no additional measurements were conducted.

The mass of excised liver samples, approximately 0.1–0.3 g, was excluded from the hepatosomatic index (HSI) measurements. The hepatosomatic index (HSI) is an indicator used to assess the liver’s relative mass in fish and is calculated as the ratio of liver mass to body mass and is expressed as a percentage [24]:

Hepatosomatic index (HSI) (%) = {Liver mass (g)/Body mass (g)} × 100

### 2.4. Hematological Analysis

Blood collection was performed at a volume of 1 mL for all samples and groups, both at the first anesthesia conducted during hepatectomy and laparotomy and at the sampling point four weeks after the start of the experiment. Blood samples were collected via the caudal vein using vacutainer tubes such as the SST (serum separation tube) and EDTA (ethylene diamine tetra-acetic acid) tube and subsequently centrifuged at 1300 g for a duration of 10 min. The following nine parameters were analyzed: alkaline phosphatase (ALP), complement activity, cortisol, glutamic oxaloacetic transaminase (GOT), glutamic pyruvic transaminase (GPT), hematocrit (Ht), hemoglobin (Hb), lysozyme activity, and total protein (TP).

The ALP (AM1055), GOT (AM103-K), GPT (AM-102), Hb (AM503), and TP (AM54-1011) measurements were performed using products from Asan Pharm, Republic of Korea, in accordance with the manufacturer’s protocol. Complement activity was determined using 100% sheep red blood cells (Innovate Research, India) and following general methods [25]. Cortisol was measured using the Cortisol ELISA kit (Enzo Life Sciences, Inc., Oyster Bay, NY, USA) according to the manufacturer’s instructions. Hematocrits were determined by transferring blood collected in EDTA tubes to heparin-treated capillary tubes (Sigma-Aldrich, St. Louis, MO, USA), which were then centrifuged at 13,000 rpm for 15 min before measurement. Lysozyme activity was measured using *Micrococcus lysodeikticus* (Sigma-Aldrich, St. Louis, MO, USA) and following general methods [26,27].

To observe hematological changes during the experimental period, blood collection was performed at the beginning of the experiment (the moment following the first anesthesia, during which hepatectomy and laparotomy were performed), and 4 weeks after the start of the experiment (the end of the experiment), six sturgeons were sampled from each group to observe the changes.

### 2.5. Histological Analysis

For histological analysis, a total of 18 organs (a total of 48 sturgeons were utilized, which were distributed into 8 groups with 6 individuals per group) were examined in this study, including the anus (hindgut), body kidney, brain, eye, gall bladder, gill, gonad, head kidney, heart, intestine, liver, muscle, pancreas, pyloric caeca, skin, spleen, stomach, and swim bladder. The groups where hepatectomy was performed were stabilized, and the excised livers were preserved, allowing for a comparative analysis of hepatic tissue conditions before and after the experiment.

Each sample was fixed in 10% neutral-buffered formalin for a duration of 24 h. Subsequently, all samples were collected and refixed in the same solution (10% neutral-buffered formalin) for an additional 24 h prior to being gradually dehydrated through the use of an ethanol series (70–100%). The samples were then cleared with xylene, embedded in paraffin, and sectioned into slices with a thickness of 4 μm. Finally, the sections were stained with hematoxylin-eosin (H&E) following standard protocols and examined under an optical microscope (Leica DM2500, Wetzlar, Germany).

### 2.6. Image and Statistics Analysis

Image analysis for the histological data was conducted using ImageJ (National Institutes of Health, Bethesda, Maryland, USA). To present histopathological data, which could potentially be subjective, as objectively as possible, image analysis was conducted using the following methods.

**Figure 2:**
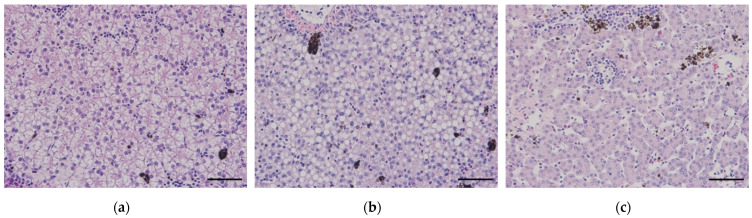
The criteria for categorizing the state of the liver tissue are as follows: (**a**) normal condition (30–45%), (**b**) fatty change (>45%), and (**c**) atrophy (<30%) (Scale bar = 50 μm).

To quantitatively distinguish the hepatic tissue’s condition, the ratio of nucleus to cell size was calculated. Ten random hepatocytes were selected from each sample, and their average ratio was used for the analysis. In the case of liver tissues, as determined by a fish pathologist, a ratio where the nucleus occupies 30–45% was defined as normal, exceeding 45% as fatty change, and below 30% as atrophy (refer to Figure 2). Additionally, the length of renal tubule epithelial cells was measured similarly. Ten random epithelial cells from proximal renal tubules were selected, and their length was measured from the basement membrane to the brush border. The average value was calculated and utilized for analysis. The hyperplasia of macrophage-like cells in the kidneys was measured by utilizing histograms to determine the proportion of activated lesions within a single image compared to the total tissue area. Randomly captured images (five in total) were used to calculate the average data for analysis. Similarly, the lymphocyte infiltration level was calculated using histograms to measure the proportion of areas occupied by lymphocytes within a single image. The entire wound area was evenly captured, and the data from each image were averaged for analysis.

The statistical analysis was performed using the SPSS 23 program (IBM, Armonk, NY, USA). Furthermore, following hepatic resection, no significant differences were observed, and the treatment groups were regrouped into the 18 °C and 28 °C groups. Prior to grouping, every individual datum was checked for statistical insignificance under the same temperature conditions. Statistical significance was assessed using one-way analysis of variance (ANOVA) and Duncan’s multiple range test, with a significance level set at (*p* < 0.05).

Due to the uneven distribution of liver tissue conditions among the groups, statistical analysis of liver status was performed using the Kruskal–Wallis H test and Scheffe post-hoc analysis. The aforementioned analysis was conducted using R (version: 4.3.0) for statistical computing.

### 2.7. Data Curation with Liver Condition

Data curation was performed on a per-group basis using image analysis, categorized according to the three conditions of hepatocytes: normal, fatty change, and atrophy (Figure 2). Furthermore, statistical analyses were conducted separately for each liver condition, even within the same low (or high) temperature group.

## 3. Results

### 3.1. Condition Factors and Hepatosomatic Index

#### 3.1.1. Condition Factor (CF)

No mortalities occurred during the experimental period, and as the experiment progressed into its fourth week, a decrease in CF values was observed throughout the entire group, including the control group (Table 1).

#### 3.1.2. Hepatosomatic Index (HSI)

Differences in HSI values were observed among the groups, with a significant decrease observed in the fourth week, particularly in the 18 °C group (including the low-temperature control) (Table 1).

### 3.2. Anesthesia and Recovery Time

The duration of anesthesia and recovery showed significant individual variation, with no significant differences observed among groups. The handling time was found to be longer in the hepatectomy group compared to the laparotomy group (naturally, the time taken for liver resection was added accordingly), and no significant difference was observed in the time required for anesthesia or recovery based on the condition of the liver tissue (Table 1).

### 3.3. Hematological Analysis

**Figure 3:**
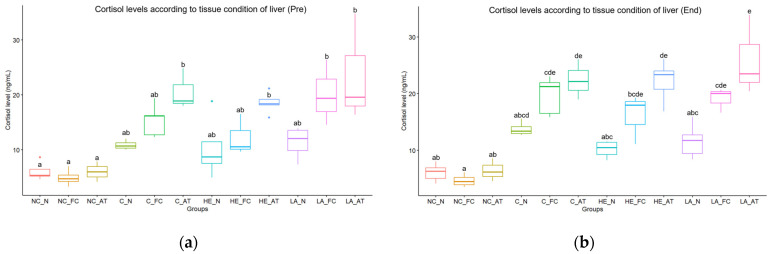
The cortisol levels according to liver condition: (**a**) cortisol levels before the start of the experiment; (**b**) cortisol levels at the end of the experiment; NC = negative control, C = control, HE = hepatectomy, LA = laparotomy, N = normal, FC = fatty change, AT = atrophy. Each box encapsulates the interquartile range, while the horizontal line within represents the median. The vertical lines extending from the box denote the maximum and minimum values. Additionally, individual data points, represented as dots, signify outliers in the dataset. The lowercase letters depicted in each graph signify inter-group statistical significance.

The results indicate that there were no statistically significant differences observed in variables other than hematocrit, hemoglobin, and cortisol between the groups. Among the individuals sampled for four consecutive weeks, the high-temperature group (including the control group) exhibited significantly higher levels of hematocrit and hemoglobin, while the low-temperature group (including the control group) displayed a significant elevation in cortisol levels (Table 2). At the termination of the experiment, specifically at the 4th week, higher levels of lysozyme activity were observed in the low-temperature hepatectomy and laparotomy groups in comparison to the other groups (Table 2). In particular, cortisol levels were found to be significantly higher in all groups that underwent anesthesia (control, hepatectomy, and laparotomy) compared to the negative control group that did not receive anesthesia. Furthermore, regardless of the presence of hepatic resection or water temperature, a significant negative correlation was observed between cortisol levels and the quality of liver tissue, indicating that cortisol levels decrease as liver tissue quality improves (Figure 3).

### 3.4. Histological Analysis

Among the 18 organs analyzed, excluding the body kidney, head kidney, liver, muscle, and spleen, no significant changes were observed (Figure S1).

No significant differences were observed between the hepatic tissues at the initiation stage of the experiment in the groups where hepatectomy was performed and the hepatic tissues at the completion stage of the experiment.

**Figure 4:**
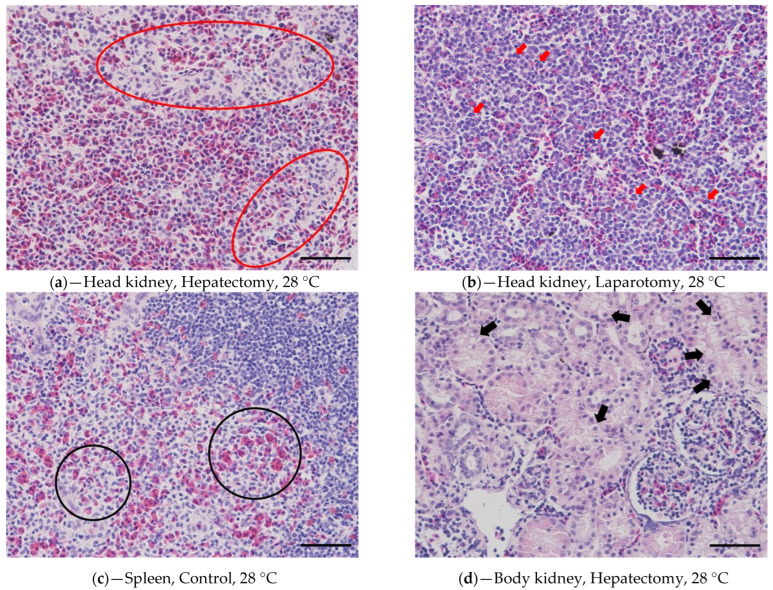

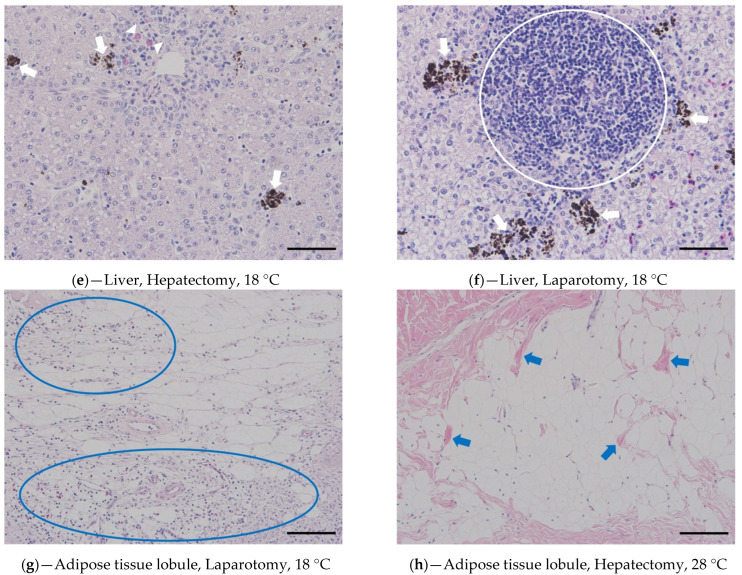
Histological results of sturgeons undergoing hepatectomy or laparotomy are presented in the following figures: (**a**,**b**) head kidney, (**c**) spleen, (**d**) body kidney, (**e**,**f**) liver, (**g**,**h**) adipose tissue lobule under wounded area; red circles = the hyperplasia of reticuloendothelial system (RES), red arrows = dividing hematopoietic cells, black circles = reticulocytes, black arrows = edema lesions, a white circle = the infiltration of inflammatory cells, white arrows = melanomacrophage centers (MMCs), blue circles = the infiltration of lymphocytes, blue arrows = the occurrence of collagen bundles (Scale bar = 50 μm).

Notably, in the head kidney, the hyperplasia of the reticuloendothelial system (RES) was observed in individuals subjected to hepatectomy and laparotomy, and this was more pronounced in the high-temperature group (Figure 4a). Moreover, an increased prevalence of actively dividing hematopoietic cells was observed in the high-temperature groups relative to the 18 °C groups (Figure 4b). In addition, immature erythrocytes (reticulocytes; irregular and eosinophilic cytoplasm was observed, differing from normal erythrocytes) were observed in the spleen of the high-temperature group, including the high-temperature control (Figure 4c). In the body kidney of the high-temperature group, including the high-temperature control, significant expansion (edema lesions) of the proximal tubule was observed (Figure 4d). In the control group, the liver displayed normal conditions, whereas the low and high-temperature groups exhibited fatty change and atrophy (Figure 4e). Furthermore, the groups that underwent surgical procedures demonstrated the infiltration of inflammatory cells (including eosinophilic granuloma cells, EGC) and an increase in melanomacrophage centers (MMCs) (Figure 4f). Obviously, leukocyte infiltration at the suture site was observed in groups that underwent hepatectomy or laparotomy (Figure 4g,h); however, in the high-temperature group, the occurrence of collagen bundles was also observed (Figure 4h). Additionally, it was noteworthy that, within the group subjected to surgical procedures, the group at 28 °C, characterized by elevated temperature, exhibited a distinctive pattern. In contrast to the 18 °C groups, where predominantly inflammatory cell infiltration in the lobular architecture of adipose tissue under the wounded area was noted (Figure 4g), the 28 °C groups displayed the presence of collagen bundles within the lobules of the subcutaneous layer of adipose tissue (Figure 4h).

### 3.5. Image Analysis

**Figure 5:**
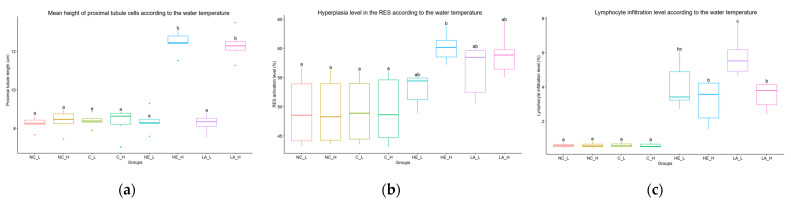
The results of image analysis are presented according to temperature and group, including (**a**) the mean height of proximal tubules, (**b**) the hyperplasia level of the reticuloendothelial system (RES), and (**c**) the level of lymphocyte infiltration; NC = negative control, C = control, HE = hepatectomy, LA = laparotomy, L = low temperature, H = high temperature. Each box encapsulates the interquartile range, while the horizontal line within represents the median. The vertical lines extending from the box denote the maximum and minimum values. Additionally, individual data points, represented as dots, signify outliers in the dataset. The lowercase letters depicted in each graph signify inter-group statistical significance.

The histological analysis of the kidneys and muscles was verified using an image analysis program (ImageJ) to ensure objectivity. The mean height of proximal tubule cells in the body kidney was significantly higher in the high-temperature group (with hepatectomy and laparotomy) (Figure 5a). The hyperplasia of the RES in the head kidney was significantly higher in the high-temperature group (with hepatectomy and laparotomy), and the low-temperature group (with hepatectomy and laparotomy) also showed a relatively higher level compared to the control group (Figure 5b). Additionally, the level of leukocyte infiltration in the wounded area was also significantly higher in the low-temperature group (with hepatectomy and laparotomy) (Figure 5c). It has been found that the differences observed in the changes occurring in the kidney and wounded area depend more on water temperature than the condition of the liver tissue (Figure 5).

## 4. Discussion

It is postulated that the reason for the decline in CF and HSI as the experiment progresses is to facilitate recovery from the damage caused by blood sampling and incision, as evidenced by the relatively lesser decrease in control compared to the hepatectomy and laparotomy groups. Furthermore, it was observed that hematocrit and hemoglobin levels did not fully recover until the 4th week in the experimental group, including the control group sampled early on. This suggests that recovery from the damage caused by blood sampling (the volume of 1 mL collected during the first anesthesia) requires more than four weeks, which warrants further investigation. However, this study has found that high temperatures can facilitate hematopoietic recovery, as demonstrated by the emergence of reticulocytes in the spleen, as well as increased levels of hematocrit and hemoglobin. This trend was observed not only in the groups where hepatectomy and laparotomy were performed but also in the high-temperature control group. As this study did not account for bleeding due to surgical procedures, further investigation is necessary to examine the changes in reticulocytes due to potential blood loss in subsequent research. Furthermore, there was an observed increase in the hematopoietic function of the head kidney [28].

In this study, high temperature yielded significant results, particularly in the context of wound healing. Specifically, a significant degree of recovery was observed in the sutured areas following hepatectomy or laparotomy compared to the low-temperature group. However, it was also noted that excessive induction of physiological activity caused damage (edema lesions) to the proximal tubules of the body’s kidney. High temperature is a type of thermotherapy used in humans and is known to induce metabolic activity [29,30].

Moreover, this study demonstrated the pivotal role of liver condition in the statistical analysis, as evidenced by variations in response observed within each group based on liver condition. No significant changes were observed in the liver tissue due to hepatectomy. Remarkably, effective management of high temperatures and liver conditions led to a significant improvement in wound healing in the fish. This can be explained by a higher degree of muscle regeneration and a lower lymphocyte infiltration index. While no significant differences in anesthesia and recovery times were observed based on liver condition, reorganizing the hematological and histological results according to liver condition revealed more notable differences, both visually and non-visually. Specifically, this study found a substantial variation in cortisol levels based on liver condition (depicted in Figure 3). Differences in cortisol levels were observed in the groups where hepatectomy and laparotomy were performed, whereas no significant differences were found in the control group. In contrast, lymphocyte infiltration in the wound site and edema lesions in the renal tubule, as well as RES activity in the head kidney, were found to be more strongly correlated with water temperature (illustrated in Figure 5).

No significant differences in both hematological and histological analyses were observed between the low- and high-temperature control groups. While this study had limitations in not accounting for variables due to differences in dissolved oxygen (DO), no statistically significant differences were observed within the control groups. This suggests that hematological and histological changes between the groups were attributed to surgical procedures rather than temperature variations. In the hematological analysis of this study, no significant changes were observed in parameters other than hematocrit, hemoglobin, and cortisol. However, one parameter that requires special attention is cortisol. In this study, a negative control without anesthesia and a control group with anesthesia were compared, and a significant difference in cortisol levels was found between the two groups. In fact, cortisol levels did not show a significant difference between the control group with anesthesia and the group subjected to surgical procedures (Table 2). This suggests that administering anesthesia itself can be a significant stressor for fish [31]. Despite the daily administration of commercial diets throughout the experimental period, a significant reduction in mass (with a confirmed decrease in CF) was observed in the group where the incision was made (Table 1), suggesting the need for a greater amount of energy for wound healing.

Overall, based on this study, it has been confirmed that surgical procedures such as hepatectomy and/or laparotomy are not harmful to the sturgeon (*Acipenser ruthenus* was used in this study) and are applicable techniques. Of particular note is the discrepancy observed in the results between Table 1 and Table 2, where differences were not apparent. This variation was dependent on the liver condition. However, further research is needed to clearly understand the stress factor increase caused by anesthesia, blood loss due to venipuncture and surgical procedures, and the time required for complete recovery of the sutured area. Additionally, to promote animal welfare and produce eco-friendly and sustainable fisheries products [1,32,33], subsequent studies must be conducted to apply surgical procedures from the perspective of animal welfare enhancement.

## 5. Conclusions

The importance of disease control to minimize mortality in fish is emphasized, and surgical techniques are discussed as an important aspect of treatment for physical injuries. Sturgeon is a suitable fish species for conducting surgical experiments due to its industrial significance and long reproductive cycle. This study focuses on investigating whether high temperatures can provide advantages in wound healing and improving physiological activity to aid in disease prevention. The use of high temperatures has yielded significant results in facilitating hematopoietic recovery and wound healing, although excessive induction of physiological activity can also cause damage. Additionally, hepatectomy and laparotomy have been confirmed as feasible surgical procedures in fish. This study revealed that anesthesia acts as a significant stressor in fish and that there are significant differences in stress response depending on the hepatic tissue status. Thus, the development of anesthesia and analgesics for improving fish welfare is necessary in the future.

## Figures and Tables

**Table 1:** Samples and experimental conditions used in this study.

Conditions		Status ^1^	Negative Control I (18 °C)	Negative Control II (28 °C)	Control I (18 °C)	Control II (28 °C)	Hepatectomy I (18 °C)	Hepatectomy II (28 °C)	Laparotomy I (18 °C)	Laparotomy II (28 °C)
Mass (g)		Pre	154.2 ± 34.7	156.6 ± 39.3	151.3 ± 28.0	160.4 ± 33.7	145.0 ± 41.6	153.7 ± 31.6	152.6 ± 37.5	145.1 ± 49.3
		End	151.4 ± 33.7	153.9 ± 38.1	146.8 ± 25.6	156.9 ± 32.8	136.0 ± 43.8	137.7 ± 28.4	146.3 ± 38.6	131.3 ± 44.5
Length (cm)		Pre	27.7 ± 2.7	27.4 ± 3.3	28.6 ± 2.8	27.9 ± 2.6	27.9 ± 2.4	28.7 ± 1.9	28.7 ± 2.2	27.6 ± 3.3
		End	27.6 ± 2.7	27.3 ± 3.3	28.6 ± 2.8	27.9 ± 2.6	28.4 ± 2.3	28.7 ± 1.9	28.9 ± 2.4	27.8 ± 3.5
Condition Factor		Pre	0.72 ± 0.05	0.76 ± 0.11	0.64 ± 0.05	0.73 ± 0.06	0.65 ± 0.04	0.64 ± 0.01	0.63 ± 0.04	0.67 ± 0.04
		End	0.72 ± 0.06	0.76 ± 0.11	0.59 ± 0.04	0.72 ± 0.06	0.58 ± 0.05	0.58 ± 0.03	0.59 ± 0.05	0.59 ± 0.05
Hepatosomatic index (%)		End	1.03 ± 0.08	0.86 ± 0.21	1.11 ± 0.20	0.97 ± 0.15	0.97 ± 0.20	0.70 ± 0.15	0.97 ± 020	0.70 ± 0.11
Temperature (°C)		-	18 ± 0.5	28 ± 0.7	18 ± 0.5	28 ± 0.7	18 ± 0.5	28 ± 0.7	18 ± 0.5	28 ± 0.7
DO * (mg/L)		-	9 ± 0.1	7.6 ± 0.1	9 ± 0.1	7.6 ± 0.1	9 ± 0.1	7.6 ± 0.1	9 ± 0.1	7.6 ± 0.1
pH		-	6.5 ± 0.1	6.5 ± 0.1	6.5 ± 0.1	6.5 ± 0.1	6.5 ± 0.1	6.5 ± 0.1	6.5 ± 0.1	6.5 ± 0.1
Time (seconds)	Anesthesia	Pre	-	-	785.2 ± 113.8	808.5 ± 62.1	731.5 ± 204.1	777.3 ± 217.7	941.2 ± 264.8	690.0 ± 153.7
		End	-	-	436.0 ± 41.2	445.3 ± 36.0	481.7 ± 121.4	749.7 ± 323.5	560.0 ± 138.3	795.8 ± 277.4
	Handling	Pre	306.7 ± 26.4	297.8 ± 26.9	305.5 ± 39.6	300.3 ± 14.6	621.5 ± 51.5	543.5 ± 17.6	492.5 ± 20.5	479.5 ± 23.8
	Recovery	Pre	-	-	1034.2 ± 295.3	828.3 ± 139.6	762.0 ± 335.6	229.5 ± 58.0	449.5 ± 182.7	263.3 ± 126.1

^1^ The start of experiment = Pre; the end of experiment = End; * DO = dissolved oxygen.

**Table 2:** Results of hematological analysis.

Parameters	Status ^1^	Negative Control I (18 °C)	Negative Control II (28 °C)	Control I (18 °C)	Control II (28 °C)	Hepatectomy I (18 °C)	Hepatectomy II (28 °C)	Laparotomy I (18 °C)	Laparotomy II (28 °C)
Hematocrits (%)	Pre	28.8 ± 3.0	30.0 ± 1.5	28.5 ± 4.4	28.2 ± 1.3	26.7 ± 2.8	29.8 ± 1.2	29.7 ± 5.8	27.2 ±3.9
	End	12.0 ± 1.4	19.0 ± 2.5	12.5 ± 1.6	19.2 ± 2.3	9.2 ± 4.2	18.2 ± 1.6	15.2 ± 2.8	19.2 ± 3.2
Hemoglobin (g/dL)	Pre	8.53 ± 1.28	8.43 ± 0.37	6.86 ± 0.45	8.39 ± 0.71	6.49 ± 0.81	8.44 ± 0.88	7.59 ± 0.69	8.04 ± 0.93
	End	4.07 ± 0.39	5.88 ± 0.73	4.07 ± 0.44	5.89 ± 1.14	3.84 ± 0.86	5.33 ± 0.58	4.91 ± 0.99	5.62 ± 0.67
Cortisol (ng/mL)	Pre	5.82 ± 1.42	5.52 ± 1.85	14.51 ± 3.29	15.78 ± 5.75	13.06 ± 5.93	15.36 ± 4.97	18.82 ± 9.50	14.41 ± 5.12
	End	6.06 ± 1.60	5.28 ± 1.64	18.54 ± 4.11	18.24 ± 5.13	13.67 ± 4.71	18.06 ± 6.76	14.53 ± 3.89	16.31 ± 6.32
ALP (U/L)	Pre	17.89 ± 1.81	18.05 ± 2.04	18.10 ± 1.75	18.21 ± 2.15	13.42 ± 2.42	13.95 ± 3.84	12.96 ± 0.98	15.24 ± 5.56
	End	10.53 ± 2.41	10.44 ± 2.06	10.67 ± 2.25	10.42 ± 2.15	9.49 ± 1.55	9.45 ± 1.36	8.36 ± 1.56	10.51 ± 2.28
Complement activity (%)	Pre	3.59 ± 1.05	3.74 ± 0.72	3.67 ± 1.07	3.58 ± 1.27	2.98 ± 0.47	3.00 ± 1.01	2.89 ± 1.00	5.08 ± 2.96
	End	5.57 ± 1.68	5.61 ± 1.58	5.75 ± 1.46	4.74 ± 0.74	4.83 ± 1.61	5.06 ± 2.06	5.02 ± 2.13	5.69 ± 2.51
GOT (U/L)	Pre	224.77 ± 18.50	224.74 ± 18.06	224.92 ± 18.20	224.75 ± 18.06	224.83 ± 39.73	217.36 ± 18.82	219.79 ± 34.45	206.51 ± 11.33
	End	191.42 ± 29.13	191.40 ± 28.86	191.24 ± 29.05	191.33 ± 28.95	182.40 ± 35.91	165.10 ± 20.05	171.91 ± 40.29	185.17 ± 37.58
GPT (U/L)	Pre	51.37 ± 14.71	51.20 ± 14.45	51.41 ± 14.59	51.12 ± 14.61	42.43 ± 14.11	31.05 ± 7.70	47.62 ± 26.18	36.21 ± 10.07
	End	20.42 ± 8.22	20.36 ± 8.18	20.46 ± 7.99	20.15 ± 8.27	21.85 ± 9.22	20.18 ± 4.50	22.16 ± 14.63	21.07 ± 5.20
Lysozyme Activity (units/mL)	Pre	2.19 ± 1.04	5.78 ± 3.12	3.64 ± 2.97	5.09 ± 3.42	3.29 ± 1.57	3.75 ± 1.84	2.87 ± 1.21	4.44 ± 1.58
	End	3.71 ± 1.09	3.73 ± 2.53	4.54 ± 2.24	3.88 ± 1.46	6.33 ± 5.09	3.54 ± 2.70	5.78 ± 3.68	2.94 ± 1.60
TP (g/dL)	Pre	0.80 ± 0.20	0.80 ± 0.19	0.79 ± 0.21	0.78 ± 0.20	0.84 ± 0.29	1.09 ± 0.23	0.93 ± 0.28	1.13 ± 0.45
	End	0.51 ± 0.09	0.52 ± 0.09	0.52 ± 0.09	0.52 ± 0.09	0.61 ± 0.25	0.47 ± 0.17	0.54 ± 0.31	0.36 ± 0.12

^1^ The start of the experiment = Pre; the end of the experiment = End.

## Data Availability

The datasets used and/or analyzed during the current study are available from the corresponding author upon reasonable request.

## Supplementary Materials

The following supporting information can be downloaded at: https://www.mdpi.com/article/10.3390/vetsci10120682/s1, Figure S1: Histological results of other organs.
