# Supplementary material for: Clinical Assessment of Thermotherapy Applications during Hepatectomy and Laparotomy in Sturgeon (Acipenser ruthenus): Impact on Bioparameter Variations Based on Liver Condition
Source: Vet Sci. 2023 Dec 1;10(12):682. doi: 10.3390/vetsci10120682 (PMC10748359; doi:10.3390/vetsci10120682)
Supplement: Supplementary file 1 [file vetsci-10-00682-s001.zip › vetsci-2686309-supplementary.pdf]

veterinary sciences

MDPI

Supplementary Materials: Figure S1: Histological results of other organs.

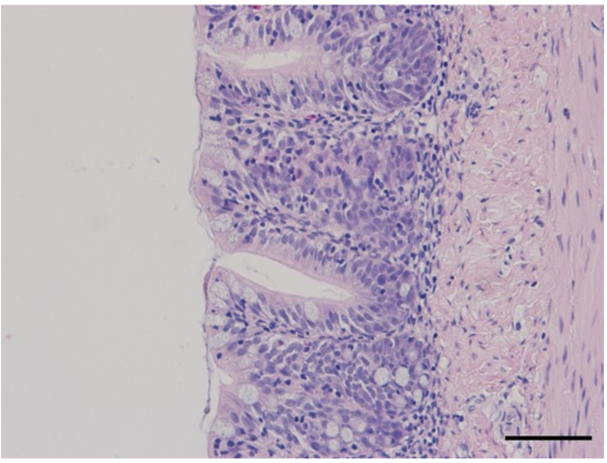

Anus (hindgut)

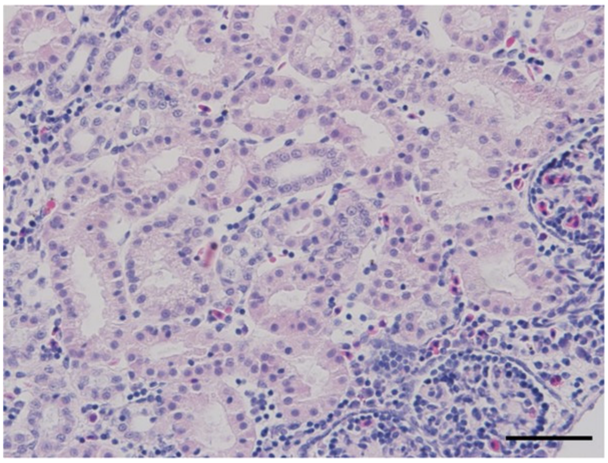

Body kidney

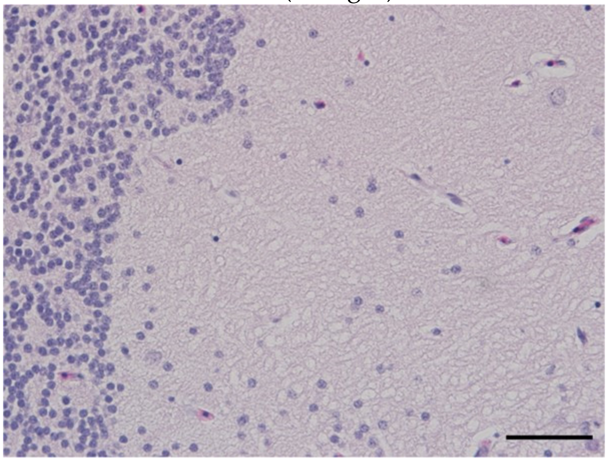

Brain (cerebellum)

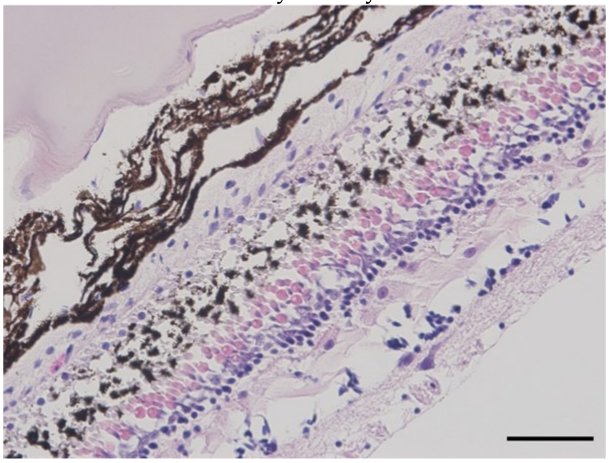

Eye

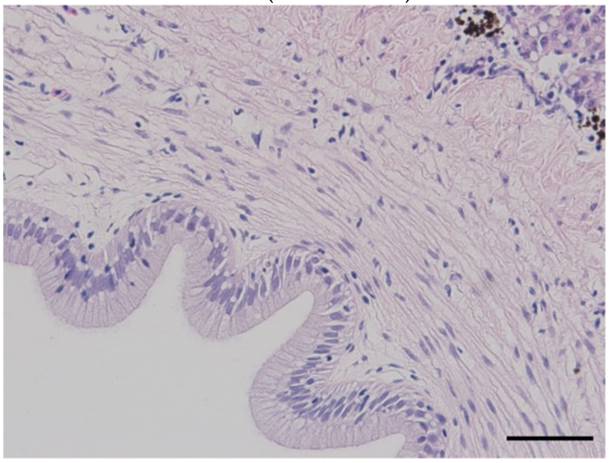

Gall bladder

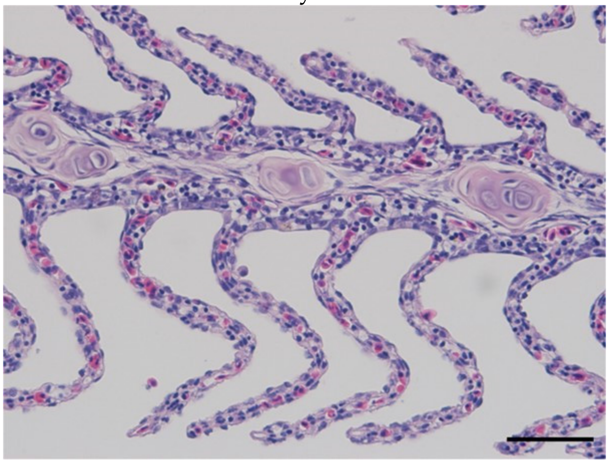

Gill

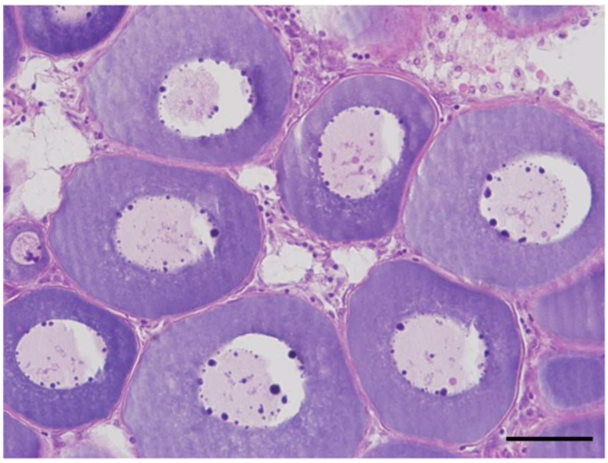

Gonad (ovary)

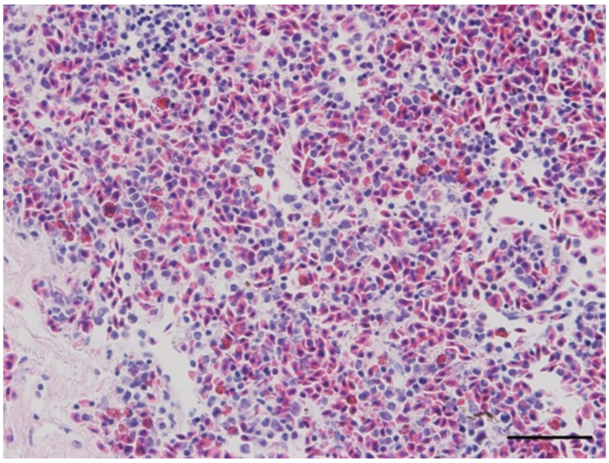

Head kidney

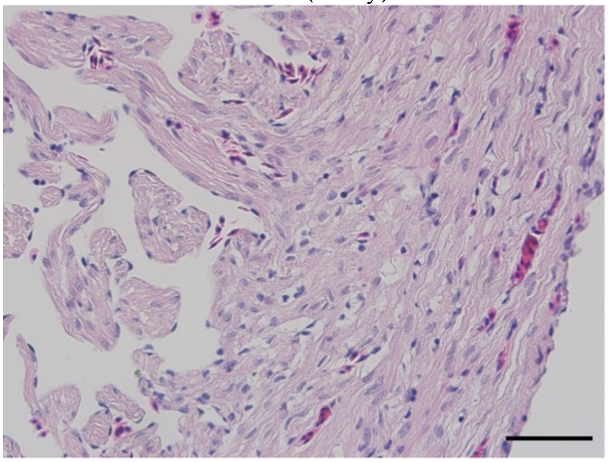

Heart

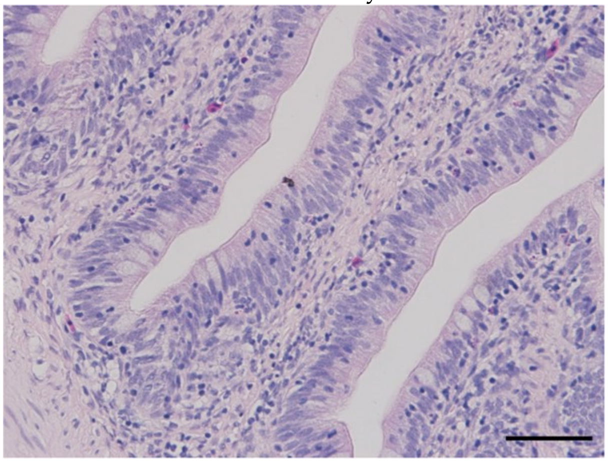

Intestine

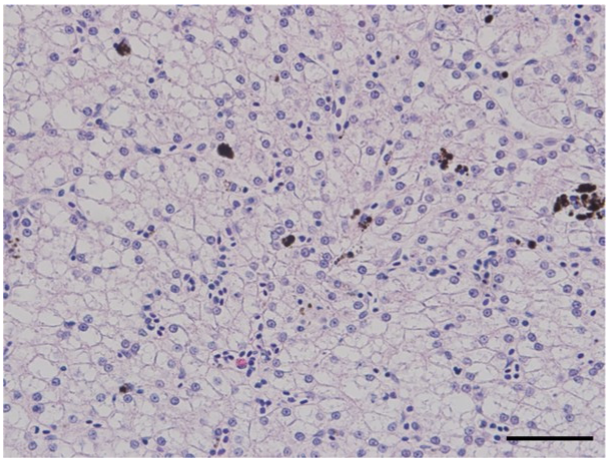

Liver

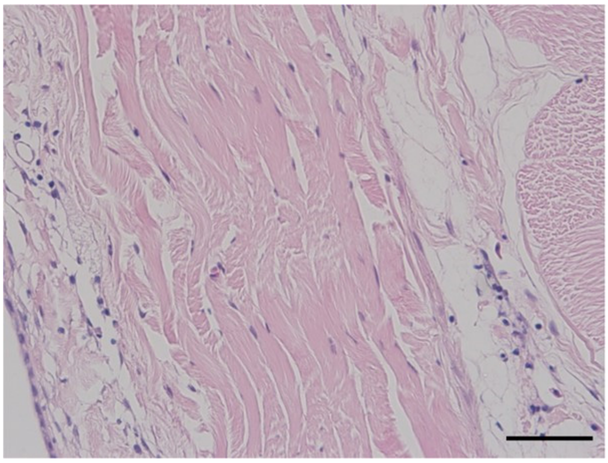

Muscle

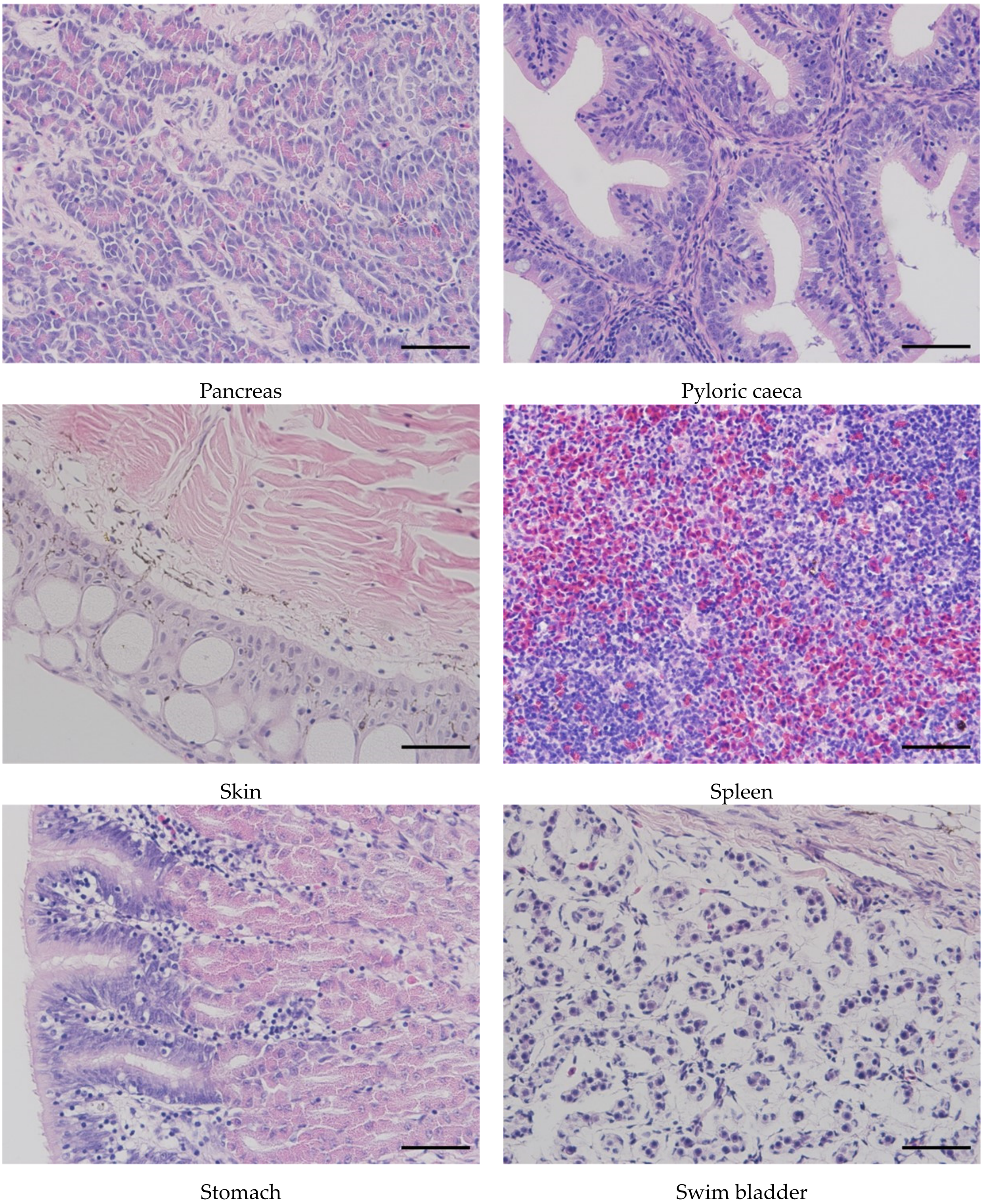

Figure S1. Other organs: anus (hindgut), body kidney, brain, eye, gall bladder, gill, gonad, head kidney, heart, intestine, liver, muscle, pancreas, pyloric caeca, skin, spleen, stomach, and swim bladder (Scale bar = 50 μm).
